# Genotype Calling from Population-Genomic Sequencing Data

**DOI:** 10.1534/g3.117.039008

**Published:** 2017-01-19

**Authors:** Takahiro Maruki, Michael Lynch

**Affiliations:** Department of Biology, Indiana University, Bloomington, Indiana 47405

**Keywords:** genotype call, polymorphism, population genomics

## Abstract

Genotype calling plays important roles in population-genomic studies, which have been greatly accelerated by sequencing technologies. To take full advantage of the resultant information, we have developed maximum-likelihood (ML) methods for calling genotypes from high-throughput sequencing data. As the statistical uncertainties associated with sequencing data depend on depths of coverage, we have developed two types of genotype callers. One approach is appropriate for low-coverage sequencing data, and incorporates population-level information on genotype frequencies and error rates pre-estimated by an ML method. Performance evaluation using computer simulations and human data shows that the proposed framework yields less biased estimates of allele frequencies and more accurate genotype calls than current widely used methods. Another type of genotype caller applies to high-coverage sequencing data, requires no prior genotype-frequency estimates, and makes no assumption on the number of alleles at a polymorphic site. Using computer simulations, we determine the depth of coverage necessary to accurately characterize polymorphisms using this second method. We applied the proposed method to high-coverage (mean 18×) sequencing data of 83 clones from a population of *Daphnia pulex*. The results show that the proposed method enables conservative and reasonably powerful detection of polymorphisms with arbitrary numbers of alleles. We have extended the proposed method to the analysis of genomic data for polyploid organisms, showing that calling accurate polyploid genotypes requires much higher coverage than diploid genotypes.

When we carry out population-genomic analyses, identifying individual genotypes is often necessary. For example, in order to identify putative loci associated with a phenotype in genome-wide association studies, calling genotypes is necessary to find which allele each individual carries at each locus. Moreover, calling individual genotypes is a first step in some population-genetic analyses. For example, many statistical methods for haplotype phasing (*e.g.*, Scheet and Stephens 2006; Browning and Browning 2007; Li *et al.* 2010) start from genotype calls at each SNP site. In addition, when accurate genotypes are called at each SNP site, traditional statistical methods, including the four-gamete test (Hudson and Kaplan 1985) and composite disequilibrium measures (Cockerham and Weir 1977; Weir 1996), can be used to examine the pattern of linkage disequilibrium.

Despite the advantages, some difficulties are associated with high-throughput sequencing technologies. One of the main difficulties is the high sequencing error rates, which typically range from 0.001 to 0.01 per read per site with commonly used sequencing platforms (Glenn 2011; Quail *et al.* 2012). Second, because sequencing occurs randomly among sites, individuals, and chromosomes in diploid organisms, depths of coverage are variable at all levels. As a result, when depths of coverage are low, there are often missing data, which introduces biases in subsequent population-genetic analyses unless they are statistically accounted for (Pool *et al.* 2010).

To call genotypes from high-throughput sequencing data, many statistical methods have been recently developed (*e.g.*, Li *et al.* 2008, 2009b; Hohenlohe *et al.* 2010; Martin *et al.* 2010; McKenna *et al.* 2010; Catchen *et al.* 2011, 2013; DePristo *et al.* 2011; Li 2011; Nielsen *et al.* 2012; Vieira *et al.* 2013). The performance of the widely used genotype callers in population-genomic analyses is not well understood, especially when the population deviates from the Hardy–Weinberg equilibrium (HWE). Recent studies (Kim *et al.* 2011; Han *et al.* 2014) found that allele frequencies estimated directly from the sequence reads are unbiased, whereas those estimated via genotype calling are biased when depths of coverage are low. These studies assumed a population in HWE. Vieira *et al.* (2013) recently showed that the performance of genotype calling can be improved by first estimating inbreeding coefficients from the sequencing data, and then calling genotypes incorporating the information on estimated inbreeding coefficients. Unfortunately, their method is applicable only when the inbreeding coefficients are non-negative (Maruki and Lynch 2015), and does not always take full advantage of the population-level information. Negative inbreeding coefficients are common in some organisms, including asexual aphids (Delmotte *et al.* 2002), *Daphnia* in permanent ponds/lakes (Hebert 1978), fruit bats (Storz *et al.* 2001), partially inbreeding plant species (Brown 1979), Laysan finches (Tarr *et al.* 1998), prairie dogs (Foltz and Hoogland 1983), rhesus monkeys (Melnick 1987), and water voles (Aars *et al.* 2006). Furthermore, some regions under balancing selection may contain excess heterozygotes and therefore show negative inbreeding coefficients (Black and Salzano 1981; Markow *et al.* 1993; Hedrick 1998; Black *et al.* 2001; Ferreira and Amos 2006; Tollenaere *et al.* 2008).

In this study, we develop a maximum-likelihood (ML) method for calling genotypes from high-throughput sequencing data that incorporates the prior information from a genotype-frequency estimator (GFE) (Maruki and Lynch 2015). We examine the performance of the proposed method using computer simulations under different genetic conditions, including those where HWE is violated, and compare the performance with that of other widely used methods. The results show that our method yields more accurate genotype calls with low or moderately high depths of coverage than the current widely used methods, which is supported by analysis of human data. In addition, we develop another ML method for calling genotypes from high-coverage sequencing data, which relaxes the assumption of biallelic polymorphisms made in many existing methods.

We also examine the necessary depth of coverage for identifying triallelic sites and accurate genotype calling with the proposed method, using computer simulations. Taking the results of the performance evaluation into account, we apply the proposed method to high-throughput sequencing data of 83 clones from a population of the microcrustacean *Daphnia pulex*, which have reasonably high depths of coverage (mean 18× per site per individual) (Lynch *et al.* 2016). The results show that the proposed method enables accurate and rapid identification of polymorphic sites with arbitrary numbers of alleles. Furthermore, we show that the proposed method can be applied to analyses of polyploid data.

## Methods

We develop ML methods for calling individual genotypes from high-throughput sequencing data. We develop two types of methods for calling genotypes, one for low-coverage sequencing data and the other for high-coverage sequencing data, because the degree of uncertainty associated with sequencing data depends on depths of coverage. In both types of methods, we statistically test the significance of polymorphisms. Also, we do not call the genotype of an individual when more than one genotype has equivalent likelihood values for the observed data.

### Genotype calling from low-coverage sequencing data

When depths of coverage are low, only one of the two parental chromosomes might be sampled, and sequence errors can resemble true variants. In such cases, the statistical uncertainties of individual sequence data can be high, thus calling accurate genotypes is not easy. However, when sequence data for multiple individuals from a population are available, the accuracy of the called genotypes can be improved by incorporating the population-level information on genotype frequencies and error rates into the genotype-calling process using Bayes’ theorem [see Martin *et al.* (2010); Nielsen *et al.* (2012); Vieira *et al.* (2013) for similar methods using the expectation-maximization algorithm for estimating priors].

Here, the genotype of an individual at a single site is called from the nucleotide read quartet (counts of A, C, G, and T) of high-throughput sequencing data at the site by an ML method. This is achieved by maximizing the likelihood of the observed data as a function of the genotype of the individual *g*. The two most abundant nucleotide reads in the population sample are considered to be candidates for alleles at the site.

Given the genotype of the individual, *g* = 1 (major homozygote, MM), 2 (heterozygote, Mm), or 3 (minor homozygote, mm), and sequencing error rate per read per site *ε*, the log-likelihood of the observed site-specific read quartet consisting of the observed counts of the most abundant (major) nucleotide read *M* (*e.g.*, C) ($n_{M}$), second most abundant (minor) nucleotide read *m* (*e.g.*, T) ($n_{m}$), and other nucleotide reads (*e.g.*, in this case A and G) ($n_{e_{1}}$ and $n_{e_{2}}$) in the population sample, $\ln L\left(n_{M},n_{m},n_{e_{1}},n_{e_{2}}|g,\epsilon \right),$ is given by the following multinomial distribution formula:

$$\ln L\left(n_{M},n_{m},n_{e_{1}},n_{e_{2}}|g,\epsilon \right)=\ln\left[\left(n!\right)/\left(n_{M}!n_{m}!n_{e_{1}}!n_{e_{2}}!\right)P_{g}{\left(M\right)}^{n_{M}}\times P_{g}{\left(m\right)}^{n_{m}}P_{g}{\left(e_{1}\right)}^{n_{e_{1}}}P_{g}{\left(e_{2}\right)}^{n_{e_{2}}}\right],$$ (1)

where $n=n_{M}+n_{m}+n_{e_{1}}+n_{e_{2}}$ (the depth of coverage). $P_{g}\left(M\right)$ is a probability of observed nucleotide read *M* with genotype *g*. It is a function of *ε* and is given by summing conditional probabilities of the observed nucleotide read, given the true nucleotide on the sequenced chromosome chosen from the pair (Table 1). The other *P* terms are similarly defined. For example, the probability of nucleotide read *M* with genotype 2 (Mm), $P_{2}\left(M\right),$ is (1/2)(1−ϵ)+(1/2)(ϵ/3), assuming the error occurs at an equal rate from the true nucleotide to one of the other three nucleotides. Because the multinomial coefficient in Equation 1 is constant regardless of the parameter values, for computational efficiency, it is ignored as follows:

**Table 1:** Probability of an observed nucleotide read as a function of the individual genotype *g* and error rate *ε*

Genotype	Nucleotide Read			
	*M*	*m*	*e*_1_	*e*_2_
1 (*MM*)	1−ϵ	ϵ/3	ϵ/3	ϵ/3
2 (*Mm*)	[(1/2)⋅(1−ϵ)]+[(1/2)⋅(ϵ/3)]	[(1/2)⋅(ϵ/3)]+[(1/2)⋅(1−ϵ)]	ϵ/3	ϵ/3
3 (*mm*)	ϵ/3	**1−ϵ**	ϵ/3	ϵ/3

*M* and *m* denote candidate alleles (the two most abundant nucleotide reads in the population sample, *e.g.*, C and T) and *e*_1_ and *e*_2_ denote other nucleotide reads (*e.g.*, in this case, A and G).

$$\ln L\left(n_{M},n_{m},n_{e_{1}},n_{e_{2}}|g,\epsilon \right)=\ln\left[P_{g}{\left(M\right)}^{n_{M}}P_{g}{\left(m\right)}^{n_{m}}P_{g}{\left(e_{1}\right)}^{n_{e_{1}}}P_{g}{\left(e_{2}\right)}^{n_{e_{2}}}\right].$$ (2)

When the genotype frequencies and error rate at the site are estimated from the population sample of nucleotide reads, they can be incorporated into Equation 2 as Bayes’ priors. We previously developed an ML method to estimate the site-specific genotype frequencies and error rate from the nucleotide read quartets (Maruki and Lynch 2015). This method yields essentially unbiased genotype-frequency estimates even with moderate depths of coverage, which we use as the Bayes’ priors necessary for improving the accuracy of genotype calls. Specifically, given the estimates of the genotype frequencies ${\hat{\gamma }}_{1}$ (frequency of major homozygotes), ${\hat{\gamma }}_{2}$ (frequency of heterozygotes), and ${\hat{\gamma }}_{3}$ (frequency of minor homozygotes), and error rate ($\hat{\epsilon }$) predetermined by the ML method of Maruki and Lynch (2015), the log-likelihood of the observed data are given as follows:

$$\ln L\left(n_{M},n_{m},n_{e_{1}},n_{e_{2}}|g,{\hat{\gamma }}_{1},{\hat{\gamma }}_{2},{\hat{\gamma }}_{3},\hat{\epsilon }\right)=\ln\left\{\left[{\hat{\gamma }}_{g}P_{g}{\left(M\right)}^{n_{M}}P_{g}{\left(m\right)}^{n_{m}}P_{g}{\left(e_{1}\right)}^{n_{e_{1}}}P_{g}{\left(e_{2}\right)}^{n_{e_{2}}}\right]/\sum_{j=1}^{3}\left[{\hat{\gamma }}_{j}P_{j}{\left(M\right)}^{n_{M}}P_{j}{\left(m\right)}^{n_{m}}P_{j}{\left(e_{1}\right)}^{n_{e_{1}}}P_{j}{\left(e_{2}\right)}^{n_{e_{2}}}\right]\right\},$$ (3)

where the ML error-rate estimate $\hat{\epsilon }$ is substituted into the *P* terms. The inferred (called) genotype of the individual is the genotype that maximizes Equation 3. To avoid calling genotypes at false polymorphic sites, we only call genotypes at significantly polymorphic sites, which are identified beforehand using the likelihood-ratio test by the population-level genotype-frequency estimator (GFE) (Maruki and Lynch 2015).

### Genotype calling from high-coverage sequencing data

When depths of coverage are high, both parental chromosomes are sequenced with high probability, and nucleotide reads derived from true variants are reliably abundant compared with those due to sequence errors. In such cases, if the confounding effects of binomial sampling of parental chromosomes and sequence errors are statistically accounted for, accurate and rapid genotype calls may be made from sequence data on each individual separately, relaxing the assumption of biallelic polymorphisms made in the method for low-coverage sequencing data and eliminating the need for prior population-level estimates of genotype frequencies.

Given the nucleotide read quartet of an individual at a site, we call the genotype of the individual by finding the genotype that maximizes the likelihood of the observed data. For example, if the nucleotide read quartet of an individual contains only A, C, and G, we examine the likelihoods of six candidate genotypes (AA, AC, AG, CC, CG, and GG). Suppose, for example, that the nucleotide read quartet of an individual contains nonzero counts for all four nucleotides. Letting $n_{A},$ $n_{C},$ $n_{G},$ and $n_{T}$ denote the counts of A, C, G, and T, respectively, the log-likelihood for genotype AA is

$$\ln L\left(n_{A},n_{C},n_{G},n_{T}|\text{AA},\epsilon \right)=\ln\left[{\left(1-\epsilon \right)}^{n_{A}}{\left(\epsilon /3\right)}^{n-n_{A}}\right],$$ (4)

where *n* is the sum of the nucleotide read counts (depth of coverage) and *ε* is the sequence error rate per read per site. This equation is a multinomial distribution formula, where the constant multinomial coefficient is ignored for computational efficiency, as in Equation 2. The subsequent likelihood functions are derived and shown in a similar way. By taking the derivative of Equation 4 with respect to *ε* and equating it to zero, *ε* is analytically estimated as

$$\hat{\epsilon }=\left(n-n_{A}\right)/n,$$ (5)

and this estimate is substituted into Equation 4 to find the likelihood for genotype AA. As another example, the log-likelihood for genotype AC is

$$\ln L\left(n_{A},n_{C},n_{G},n_{T}|\text{AC},\epsilon \right)=\ln\left[{\left(1/2-\epsilon /3\right)}^{n_{A}+n_{C}}{\left(\epsilon /3\right)}^{n_{G}+n_{T}}\right],$$ (6)

where *ε* is similarly estimated as

$$\hat{\epsilon }=\left(3/2\right)\cdot \left[\left(n_{G}+n_{T}\right)/n\right],$$ (7)

and again, this estimate is substituted into Equation 6 to find the likelihood for genotype AC. The likelihoods for the other eight candidate genotypes are similarly calculated.

### Statistical tests of called genotypes in the high-coverage genotype caller

Because of high sequencing error rates, genotypes called by the high-coverage genotype caller (HGC) might sometimes falsely suggest polymorphisms. To minimize analyzing false polymorphisms and misidentifying sites containing three or four alleles, we examine statistical significance of called genotypes by likelihood-ratio tests (Kendall and Stuart 1979). Specifically, we examine the statistical significance of called genotypes with respect to the genotype homozygous for the most abundant nucleotide in the population sample *M* (MM).

Under the null hypothesis of monomorphism, the site is fixed for *M*. Under the alternative hypothesis of polymorphism, at least one individual has a genotype different from MM. Therefore, we reject the null hypothesis of population monomorphism if the likelihood of at least one non-MM called genotype is significantly greater than that of the MM genotype. Specifically, letting $LL_{0}$ and $LL_{1}$ denote the log-likelihoods of the observed data of an individual for the MM genotype and that for a non-MM called genotype, the likelihood-ratio test statistic (*LRT*) for the individual is

$$LRT=2\left(LL_{1}-LL_{0}\right).$$ (8)

This test statistic is expected to be asymptotically $\chi^{2}$-distributed with one degree of freedom. We reject the null hypothesis of population monomorphism when LRT is significant for at least one of the non-MM called genotypes at a user-specified level.

We estimate the number of alleles by examining the nucleotides contained in significant genotypes. To minimize misidentification of sites containing three or four alleles, when the significant genotype is heterozygous, we compare the likelihood of the called genotype to that of the genotype homozygous for the more abundant nucleotide in the individual, and consider the genotype heterozygous only if the former is significantly greater than the latter at the specified level by the likelihood-ratio test with one degree of freedom. Otherwise, the genotype is considered homozygous for estimating the number of alleles.

### Genotype calling from triploid sequencing data

The HGC explained above can be extended to triploid data. Specifically, assuming that sequencing occurs randomly among the three chromosomes, and equal error rates occur from the true nucleotide to one of the other three, the probability of an observed nucleotide read as a function of the genotype of an individual is found in a way analogous to that for diploid data (Supplemental Material, Table S1). Then, the likelihood of the observed nucleotide read quartet of an individual can be formulated. As with diploid data, we choose the genotype that maximizes the likelihood to call the genotype of the individual. The proposed method can be applied to low-coverage sequencing data, although high coverage is needed to call accurate genotypes.

As an example, suppose that the nucleotide read quartet of an individual contains nonzero counts for all four nucleotides. Letting $n_{A},$ $n_{C},$ $n_{G},$ and $n_{T}$ denote the counts of A, C, G, and T, respectively, the log-likelihood for genotype AAA is

$$\ln L\left(n_{A},n_{C},n_{G},n_{T}|\text{AAA},\epsilon \right)=\ln\left[{\left(1-\epsilon \right)}^{n_{A}}{\left(\epsilon /3\right)}^{n-n_{A}}\right],$$ (9)

where *n* is the sum of the nucleotide read counts (depth of coverage), and *ε* is the sequence error rate per read per site. By taking the derivative of Equation 9 with respect to *ε* and equating it to zero, *ε* is analytically estimated as

$$\hat{\epsilon }=\left(n-n_{A}\right)/n,$$ (10)

and this estimate is substituted into Equation 9 to find the likelihood for genotype AAA. As another example, the log-likelihood for genotype ACC is

$$\ln L\left(n_{A},n_{C},n_{G},n_{T}|\text{ACC},\epsilon \right)=\ln\left\{{\left[\left(\text{1}/\text{3}\right)-\left(\epsilon /9\right)\right]}^{n_{A}}{\left[\left(2/3\right)-\left(5/9\right)\cdot \epsilon )\right]}^{n_{C}}{\left(\epsilon /3\right)}^{n_{G}+n_{T}}\right\},$$ (11)

where *ε* is estimated as

$$\hat{\epsilon }=\left[6n+9n_{C}+15n_{G}+15n_{T}-\sqrt{{\left(6n+9n_{C}+15n_{G}+15n_{T}\right)}^{2}-360n\left(n_{G}+n_{T}\right)}\right]/10n,$$ (12)

and again, this estimate is substituted into Equation 11 to find the likelihood for genotype ACC. As a final example, the log-likelihood for genotype ACG is

$$\ln L\left(n_{A},n_{C},n_{G},n_{T}|\text{ACG},\epsilon \right)=\ln\left[{\left(1/3-\epsilon /9\right)}^{n_{A}+n_{C}+n_{G}}{\left(\epsilon /3\right)}^{n_{T}}\right],$$ (13)

where *ε* is estimated as

$$\hat{\epsilon }=3\cdot \left[\left(n_{T}\right)/n\right],$$ (14)

and again, this estimate is substituted into Equation 13 to find the likelihood for genotype ACG. To avoid analyzing false polymorphisms due to sequencing errors, the statistical significance of called genotypes is examined by likelihood-ratio tests analogous to those with diploid data, and the number of alleles is based on significant genotypes.

### Genotype calling from tetraploid sequencing data

Because tetraploid species are common in plants and some animals, we also formulate likelihood functions for a tetraploid genotype caller. Assuming that sequencing occurs randomly among the four chromosomes, and equal error rates occur from the true nucleotide to one of the other three, the probability of an observed nucleotide read as a function of the genotype of an individual is similarly found (Table S2). As with triploid data, we find the genotype that maximizes the likelihood of the observed nucleotide read quartet of an individual to call the genotype of the individual.

As an example, suppose that the nucleotide read quartet of an individual contains nonzero counts for all four nucleotides. Letting $n_{A},$ $n_{C},$ $n_{G},$ and $n_{T}$ denote the counts of A, C, G, and T, respectively, the log-likelihood for genotype AAAA is

$$\ln L\left(n_{A},n_{C},n_{G},n_{T}|\text{AAAA},\epsilon \right)=\ln\left[{\left(1-\epsilon \right)}^{n_{A}}{\left(\epsilon /3\right)}^{n-n_{A}}\right],$$ (15)

where *n* is the sum of the nucleotide read counts (depth of coverage) and *ε* is the sequence error rate per read per site. By taking the derivative of Equation 15 with respect to *ε* and equating it to zero, *ε* is analytically estimated as

$$\hat{\epsilon }=\left[\left(n-n_{A}\right)/n\right],$$ (16)

and this estimate is substituted into Equation 15 to find the likelihood for genotype AAAA. As another example, the log-likelihood for genotype ACCC is

$$\ln L\left(n_{A},n_{C},n_{G},n_{T}|\text{ACCC},\epsilon \right)=\ln\left\{{\left(1/4\right)}^{n_{A}}{\left[\left(3/4\right)-\left(2/3\right)\cdot \epsilon )\right]}^{n_{C}}{\left(\epsilon /3\right)}^{n_{G}+n_{T}}\right\},$$ (17)

where *ε* is estimated as

$$\hat{\epsilon }=\left(9/8\right)\cdot \left[\left(n_{G}+n_{T}\right)/\left(n_{C}+n_{G}+n_{T}\right)\right],$$ (18)

and again, this estimate is substituted into Equation 17 to find the likelihood for genotype ACCC. As another example, the log-likelihood for genotype AACC is

$$\ln L\left(n_{A},n_{C},n_{G},n_{T}|\text{AACC},\epsilon \right)=\ln\left\{{\left[\left(1/2\right)-\left(\epsilon /3\right)\right]}^{n_{A}+n_{C}}{\left(\epsilon /3\right)}^{n_{G}+n_{T}}\right\},$$ (19)

where *ε* is estimated as

$$\hat{\epsilon }=\left(3/2\right)\cdot \left[\left(n_{G}+n_{T}\right)/n\right],$$ (20)

and again, this estimate is substituted into Equation 19 to find the likelihood for genotype AACC. As a final example, the log-likelihood for genotype AACG is

$$\ln L\left(n_{A},n_{C},n_{G},n_{T}|\text{AACG},\epsilon \right)=\ln\left\{{\left[\left(1/2\right)-\left(\epsilon /3\right)\right]}^{n_{A}}{\left(1/4\right)}^{n_{C}+n_{G}}{\left(\epsilon /3\right)}^{n_{T}}\right\},$$ (21)

where *ε* is estimated as

$$\hat{\epsilon }=\left(3/2\right)\cdot \left[n_{T}/\left(n_{A}+n_{T}\right)\right],$$ (22)

and again, this estimate is substituted into Equation 21 to find the likelihood for genotype AACG. Again, we report the number of alleles at a site based on significant genotypes to avoid analyzing false polymorphisms due to sequencing errors.

### Generation of diploid nucleotide read data at biallelic sites using computer simulations

To examine the performance of the genotype callers at biallelic sites, we generated nucleotide read data for *N* diploid individuals by computer simulations and called individual genotypes from the simulated data. In the simulations, the probability of sampling an individual with a particular genotype was equal to its relative frequency in the population. The population frequencies of major and minor homozygotes were specified by $\gamma_{1}$ and $\gamma_{3},$ respectively. To compare the called genotypes with the true genotypes, we recorded the true genotype of each individual. The depths of coverage were assumed to be Poisson-distributed with mean *μ* among the individuals, and were specified as

$$c\left(X,\mu \right)=\left[{\left(\mu \right)}^{X}e^{-\mu }\right]/X!,$$ (23)

where *X* is a particular value of the coverage for an individual and *c* is a probability mass function of *X*. The nucleotide reads from each individual were randomly chosen from its genotype allowing for errors. Sequence errors were randomly introduced at rate *ε* per read per site from the true nucleotide to one of the other three nucleotides.

### Comparison of the Bayesian genotype caller with other widely used methods

To compare the performance of our Bayesian genotype caller (BGC) with that of other widely used methods for calling genotypes, we called individual genotypes using GATK (version 3.4-0) (McKenna *et al.* 2010; DePristo *et al.* 2011; Van der Auwera *et al.* 2013) and Samtools (version 0.1.19) (Li 2011) from the same simulated nucleotide read data generated by the method described above, and compared the accuracy of the genotypes called by different methods. Although both GATK and Samtools use Bayesian genotype-frequency priors, GATK uses the same priors for all sites and Samtools assumes HWE, and therefore their approaches differ from ours. In addition to these genotype-calling methods, we also compared our performance with that of the corresponding genotype-calling function in ANGSD (version 0.911) (Korneliussen *et al.* 2014), which is also designed especially for low-coverage sequencing data. To make the generated data applicable to all methods, including ours, we made BAM files of sequence read data mapped to a simulated reference sequence.

First, we generated a simulated reference sequence consisting of random nucleotides. Each of a total of 10,000 biallelic sites was surrounded by 50 fixed nucleotides on both sides, so that sequence reads of 101 bp are uniquely mapped to the reference sequence. We outputted the 101-bp sequence reads in the FASTA format with nucleotide reads at biallelic sites characterized in the manner described above. The sequence reads were mapped to the reference sequence using Novoalign (version 3.00.02) (www.novocraft.com). We converted SAM files of mapped reads to BAM files using Samtools (Li *et al.* 2009a).

To call genotypes with GATK, we made a dictionary file of the reference sequence and added read groups to the BAM files using Picard (version 1.134) (http://broadinstitute.github.io/picard). Then, we sorted and indexed the BAM files using Samtools. We generated individual GVCF files, which contain information of called genotypes, from the BAM files using HaplotypeCaller, which individually calls genotypes, assigning an arbitrarily chosen uniform base quality score (30) to every base. Changing the base quality score, however, from 30 to 20, did not noticeably influence the results (results not shown). Then, we called genotypes from the GVCF files using Genotype_GVCFs, which refines the called genotypes using the population-level information. We called genotypes with Samtools from the BAM files using mpileup and bcftools to use the population-level information. To call genotypes with ANGSD, we first converted the BAM files to SAM files using Samtools. Then, we assigned the same uniform base quality score of 30 to every base, using a custom Perl script. The resulting SAM files with base quality scores were converted back to BAM files as input for ANGSD, using Samtools. We used a Bayesian genotype-calling method corresponding to ours in ANGSD, setting the value of the -doPost option at one, using Samtools genotype likelihoods, and setting the statistical significance for calling SNPs at the 5% level. Unlike other methods, our method does not rely just on read quality, and estimates error rates from sequence data themselves. This unique feature of our method is important because errors can result from other factors including those introduced during sample preparation.

The ML estimates of genotype frequencies and error rates, necessary for calling genotypes with BGC, were obtained using GFE (available from https://github.com/Takahiro-Maruki/Package-GFE; an updated version with a function to prepare the input file of the BGC and its documentation are available as File S1 and File S2) (Maruki and Lynch 2015). The individual pro files of nucleotide read quartets were generated using sam2pro (version 0.6) (http://guanine.evolbio.mpg.de/mlRho/sam2pro_0.6.tgz) from mpileup files generated from the BAM files by Samtools.

In addition to examining the performance of genotype calling, we compared the allele-frequency estimates by GFE to those by other methods. The allele-frequency estimates via genotype calling by GATK and Samtools were calculated from the VCF files using VCFtools (version 0.1.11) (Danecek *et al.* 2011). The allele-frequency estimates by ANGSD were estimated directly from sequence reads by the method of Kim *et al.* (2011).

To examine how different genotype-calling methods perform when applied to real sequencing data, we applied them to low-coverage (mean 7.6× per site per individual) sequencing data of the phase 3 1000 Genomes project (1000 Genomes Project Consortium 2015) on chromosome 11 in the CEU population. To assess the accuracy of genotype calls by different methods, we compared the called genotypes with the corresponding genotypes in phase II and III data of the International HapMap project (International HapMap Consortium 2007; International HapMap 3 Consortium 2010). Specifically, we downloaded the BAM files of the phase 3 1000 Genomes sequencing data in 99 CEU individuals on chromosome 11 from ftp.1000genomes.ebi.ac.uk/vol1/ftp/phase3/data/. We also downloaded the FASTA file of the reference sequence (human_g1k_v37.fasta) from ftp.1000genomes.ebi.ac.uk/vol1/ftp/technical/reference/. We downloaded the corresponding genotypes in the HapMap phase II and III data from ftp.ncbi.nlm.nih.gov/hapmap/genotypes/2010-08_phaseII+III/forward/. We found that 94 out of the 99 CEU individuals had both 1000 Genomes and HapMap data, and therefore used the 94 individuals with the necessary data in the subsequent analyses.

To prepare high-quality input data for calling genotypes, we marked duplicate reads and locally realigned sequences around indels in the BAM files using GATK (version 3.4-0) (McKenna *et al.* 2010; DePristo *et al.* 2011) and Picard (version 1.107) (http://broadinstitute.github.io/picard), following GATK best practices (Van der Auwera *et al.* 2013). In addition, we clipped overlapping read pairs in the BAM files using BamUtil (version 1.0.13) (http://genome.sph.umich.edu/wiki/BamUtil). We made mpileup files of the 94 individuals from the processed BAM files using Samtools. The pro files of nucleotide read quartets were made from the mpileup files using sam2pro (version 0.8) (http://guanine.evolbio.mpg.de/mlRho/sam2pro_0.8.tgz). The file of nucleotide read quartets of 94 individuals necessary for the proposed method was made from the pro files using GFE (Maruki and Lynch 2015).

To call genotypes with GATK, we first sorted and indexed the BAM files using Samtools. Next, we generated individual GVCF files from the BAM files using HaplotypeCaller. Then, we called genotypes from the GVCF files using Genotype_GVCFs. We called genotypes with Samtools from the BAM files using mpileup and bcftools using the population-level information. To call genotypes with ANGSD from the BAM files, we used a Bayesian genotype-calling method corresponding to ours in ANGSD setting the value of the -doPost option at one, using Samtools genotype likelihoods and setting the statistical significance for calling SNPs at the 5% level. In addition to these genotype-calling methods, we examined how the popular genotype-imputation method performs by imputing missing genotypes in GATK calls using Beagle (version 4.1) (Browning and Browning 2016). Because preexisting high-quality reference panels (genotypes/haplotypes) do not exist in the vast majority of the organisms, except a few model organisms such as humans and flies, we imputed the genotypes without a reference panel. Here, we imputed genotypes using genotype likelihoods instead of called genotypes to take the uncertainty of genotype calls into account.

We calculated the correct-call rate among individuals as a fraction of individuals with genotype calls identical to HapMap genotypes, assuming that HapMap genotypes are correct. To examine the accuracy of actually called genotypes, we also calculated the correct-call rate among called genotypes, where the fraction is calculated among called genotypes. We converted the NCBI build 36 coordinates in the HapMap data to the GRCh37 coordinates in the 1000 Genomes data using the UCSC liftOver (Speir *et al.* 2016) to make the coordinates in the two data sets consistent with each other. To minimize the confounding effect of mismapping, we excluded sites involved in putative repetitive regions identified by RepeatMasker (http://www.repeatmasker.org/) and those with population coverage (sum of the coverage over the individuals) less than half the mean or greater than one and a half means from the analyses. We downloaded the repeat-masked reference sequence from the Ensembl website (ftp.ensembl.org/pub/release-75/fasta/homo_sapiens/dna/) to identify sites involved in putative repetitive regions.

### Performance evaluation of the HGC as a function of coverage

Because of the random sampling of parental chromosomes, only one of the two chromosomes might be sampled when the depth of coverage for an individual is low. Therefore, the ability of a genotype caller to correctly infer individual genotypes is mainly limited by its ability to call heterozygotes when the depth of coverage is low. In addition, when the depth of coverage is low, homozygotes can appear heterozygous due to sequencing errors. Therefore, examining the effect of coverage on the accuracy of called genotypes is important for finding the optimal sequencing strategy for genotype ascertainment.

To examine how much coverage is needed to accurately call genotypes using the HGC, we examined its rates for correctly calling homozygotes and heterozygotes as functions of the depth of coverage. Specifically, we generated nucleotide read data from an individual having a homozygous or heterozygous genotype with a fixed depth of coverage and error rate *ε*, and called the genotype of the individual with HGC. We repeated this process for 10,000 simulation replications and calculated the correct-call rate as a fraction of simulation replications where the genotype was correctly called by HGC.

### Performance evaluation of genotype callers at triallelic sites using computer simulations

To examine the performance of the HGC for identifying sites with more than two alleles and calling genotypes in population samples, we generated BAM files of sequence read data in a way similar to that at biallelic sites, where nucleotide read data at each of a total of 10,000 triallelic sites for *N* diploid individuals were generated, according to their genotype frequencies, introducing errors at rate *ε*. Here, for simplicity, we specified the population genotype frequencies by assigning population allele frequencies *p*, *q*, and *r* to the most abundant, second most abundant, and the rarest allele, respectively, and assuming HWE, although our method makes no assumption on the mating system. As GATK and Samtools are both capable of calling genotypes at triallelic sites, we applied them to the BAM files using HaplotypeCaller and GenotypeGVCF, and mpileup and bcftools with the -m option, respectively, and compared their correct-call rates to that of HGC. In addition, we applied our BGC to the BAM files and examined the corresponding correct-call rate to find the effect of incorrectly assuming at most two alleles on the accuracy of genotype calls at triallelic sites.

### Application of the HGC to empirical data

To examine the performance of the HGC with real data, we applied it to high-throughput Illumina sequencing data of 83 *D. pulex* clones from Kickapond (Lynch *et al.* 2016). We mapped the sequencing data to the PA42 reference genome (version 3.0) (Z. Ye, S. Xu, K. Spitze, J. Asselman, X. Jiang, M. E. Pfrender, and M. Lynch, unpublished results) using Novoalign (version 3.02.11) (www.novocraft.com) with the “-r None” option to prevent it from mapping a read if it matched more than one location. We converted the SAM files of mapped sequencing data to BAM files using Samtools (version 0.1.18) (Li *et al.* 2009a). Then, we marked duplicate reads and locally realigned sequences around indels using GATK (version 3.4-0) (McKenna *et al.* 2010; DePristo *et al.* 2011) and Picard (version 1.107) (http://broadinstitute.github.io/picard), following GATK best practices (Van der Auwera *et al.* 2013). In addition, we clipped overlapping read pairs using BamUtil (version 1.0.13) (http://genome.sph.umich.edu/wiki/BamUtil). We made mpileup files of the 83 clones from the processed BAM files using Samtools. The pro files of nucleotide read quartets were made from the mpileup files using sam2pro (version 0.8) (http://guanine.evolbio.mpg.de/mlRho/sam2pro_0.8.tgz). The input file of nucleotide read quartets of 83 clones was made from the pro files using GFE (Maruki and Lynch 2015). To avoid analyzing misassembled regions, we excluded regions considered to be misassembled by REAPR (version 1.134) (Hunt *et al.* 2013) from our analyses. We set the minimum coverage required to call a genotype of an individual at six. To minimize analyzing sites with data on a small number of individuals or those with mismapped reads, we only analyzed sites with the population coverage (sum of the coverage over the individuals) at least half the mean and at most 1.5× of the mean. Furthermore, we excluded sites involved in putative repetitive regions identified by RepeatMasker (version 4.0.5) (http://www.repeatmasker.org/) with the RepeatMasker library (Jurka *et al.* 2005) made on August 7, 2015. In addition, to further reduce analyzing sites with mismapped reads, we excluded sites with mean error-rate estimates among clones of >0.01.

### Performance evaluation of the triploid genotype caller as a function of coverage

To examine the effect of coverage on the accuracy of genotypes called by the triploid genotype caller (TRI), we examined the correct-call rate as a function of the depth of coverage in a way similar to that for diploid data. Here, we examined the correct-call rate for three different types of genotypes (homozygotes, heterozygotes containing two different nucleotides, and heterozygotes containing three different nucleotides) among a total of 10,000 simulation replications.

### Comparison of the TRI with GATK

To compare the performance of the TRI to that of other widely used methods, we applied our TRI and GATK (version 3.4-0) (McKenna *et al.* 2010; DePristo *et al.* 2011) to BAM files of simulated sequence read data generated in a way similar to that for diploid data, and compared the correct-call rates. Here, we generated fixed-coverage read data from an individual with a particular genotype 10,000 times. To call triploid genotypes with GATK, we set the ploidy of HaplotypeCaller at three.

### Data availability

Source codes, written in C++, implementing the proposed methods and their documentation are available as supporting information (File S1, File S2, File S3, File S4, File S5, File S6, File S7, File S8, File S9, File S10, File S11, and File S12).

## Results

The performance of the BGC when applied to low-coverage sequencing data at biallelic sites was evaluated with simulated data and compared with the corresponding performance of GATK, Samtools, and ANGSD. In addition, the allele-frequency estimates by the GFE were compared with those via genotype calling by GATK and Samtools and those by a method of Kim *et al.* (2011), as implemented in ANGSD. To examine the performance under the worst situation, the error rate was set at 0.01, which is typically the upper bound with commonly used sequencing platforms. We evaluated the performance under three different genetic conditions, where the inbreeding coefficient *f* was zero (HWE), minimized, or maximized given minor-allele frequency *q*. When *f* is minimized, the frequencies of major homozygotes and minor homozygotes, $\gamma_{1}$ and $\gamma_{3},$ are 1−2q and 0, respectively. When *f* is maximized, $\gamma_{1}$ and $\gamma_{3}$ are 1−q and *q*, respectively.

When the depth of coverage is low (mean 3×), the allele-frequency estimates by GFE are unbiased, whereas those via genotype calling are biased under all examined conditions (Table 2). The allele-frequency estimates by ANGSD are similar to ours, although they are slightly biased when *f* is minimized or maximized. The allele-frequency estimates via genotype calling by GATK are more biased than those by Samtools when *f* is zero or minimized. On the other hand, when *f* is maximized, the allele-frequency estimates via genotype calling by Samtools are more biased than those by GATK. The correct-call rate among individuals by the proposed method (BGC) is higher than that by GATK, and lower than that by Samtools and ANGSD when *f* is zero or minimized. When *f* is maximized, the correct-call rate by BGC is the highest. The highest correct-call rate among individuals by Samtools and ANGSD when *f* is zero or minimized is mainly because both methods always call individual genotypes regardless of the depth of coverage, even when there is no read. In fact, the correct-call rate among called genotypes by BGC is the highest under the majority of the examined conditions.

**Table 2:** Comparison of the allele-frequency estimates and called genotypes by different methods with low depths of coverage

Method	*q*	*f*	$\hat{q}$ (mean ± 2 SEM)	Correct-Call Rate among Individuals (mean ± 2 SEM)	Correct-Call Rate among Called Genotypes (mean ± 2 SEM)
Proposed	0.1	0 (HWE)	0.10 ± 0.00050	0.84 ± 0.00064	0.91 ± 0.00049
GATK	0.1	0 (HWE)	0.06 ± 0.00042	0.80 ± 0.00073	0.88 ± 0.00057
Samtools	0.1	0 (HWE)	0.08 ± 0.00039	0.90 ± 0.00045	0.90 ± 0.00045
ANGSD	0.1	0 (HWE)	0.10 ± 0.00050	0.90 ± 0.00045	0.90 ± 0.00045
Proposed	0.1	Minimized	0.10 ± 0.00050	0.87 ± 0.00068	0.95 ± 0.00049
GATK	0.1	Minimized	0.06 ± 0.00041	0.82 ± 0.00077	0.91 ± 0.00061
Samtools	0.1	Minimized	0.08 ± 0.00039	0.94 ± 0.00047	0.94 ± 0.00047
ANGSD	0.1	Minimized	0.11 ± 0.00050	0.94 ± 0.00046	0.94 ± 0.00046
Proposed	0.1	Maximized	0.10 ± 0.00063	0.95 ± 0.00046	1.00 ± 0.00015
GATK	0.1	Maximized	0.09 ± 0.00058	0.93 ± 0.00051	1.00 ± 0.00006
Samtools	0.1	Maximized	0.06 ± 0.00046	0.91 ± 0.00047	0.91 ± 0.00047
ANGSD	0.1	Maximized	0.08 ± 0.00057	0.91 ± 0.00047	0.91 ± 0.00047
Proposed	0.3	0 (HWE)	0.30 ± 0.00077	0.77 ± 0.00081	0.88 ± 0.00080
GATK	0.3	0 (HWE)	0.22 ± 0.00077	0.68 ± 0.00094	0.79 ± 0.00088
Samtools	0.3	0 (HWE)	0.25 ± 0.00096	0.84 ± 0.00074	0.84 ± 0.00074
ANGSD	0.3	0 (HWE)	0.30 ± 0.00076	0.84 ± 0.00074	0.84 ± 0.00074
Proposed	0.3	Minimized	0.30 ± 0.00063	0.74 ± 0.00087	0.87 ± 0.00075
GATK	0.3	Minimized	0.19 ± 0.00067	0.58 ± 0.00098	0.70 ± 0.00101
Samtools	0.3	Minimized	0.26 ± 0.00085	0.83 ± 0.00078	0.83 ± 0.00078
ANGSD	0.3	Minimized	0.31 ± 0.00064	0.83 ± 0.00078	0.83 ± 0.00078
Proposed	0.3	Maximized	0.30 ± 0.00094	0.94 ± 0.00047	1.00 ± 0.00017
GATK	0.3	Maximized	0.26 ± 0.00093	0.90 ± 0.00061	1.00 ± 0.00011
Samtools	0.3	Maximized	0.24 ± 0.00118	0.87 ± 0.00067	0.87 ± 0.00067
ANGSD	0.3	Maximized	0.28 ± 0.00102	0.87 ± 0.00067	0.87 ± 0.00067

*q*, $\hat{q},$ and *f* are the minor-allele frequency, its estimate, and inbreeding coefficient, respectively. $\hat{q}$ by the proposed method and ANGSD are directly estimated from sequence read data by the genotype-frequency estimator (Maruki and Lynch 2015) and Kim *et al.*’s (2011) method, respectively. Called genotypes by the proposed method are by the Bayesian genotype caller. The correct-call rate among individuals is a fraction of individuals with correctly called genotypes among *N* = 100 individuals, where missing genotype calls are considered incorrect. On the other hand, the correct-call rate among called genotypes is calculated only among individuals with called genotypes. Mean depth of coverage *µ* = 3, error rate *ε* = 0.01. Results are based on a total of 10,000 simulation replications for each parameter set. HWE, Hardy–Weinberg equilibrium.

When the depth of coverage is moderately high (mean 10×), the allele-frequency estimates by all methods are similar to each other and nearly unbiased under all examined conditions (Table S3). Consistent with this, the correct-call rates by all methods are high under all examined conditions. However, the correct-call rate by BGC is the highest under all examined conditions. We confirmed that we correctly generated the simulated data by examining the realized parameter values in the population samples (Table S4).

Our conclusions on the performance of the genotype-calling methods based on computer simulations are further supported by qualitatively similar results of the corresponding performance evaluation using human data (Table 3). Similar to the simulation-based results of the performance evaluation when the population is in HWE, the correct-call rate among individuals of BGC was lower than that by ANGSD and Samtools and higher than that by GATK. Again, this is mainly because the former two methods call individual genotypes even when there is no read, and the correct-call rate among called genotypes by BGC is the highest. The correct-call rate among individuals of the Beagle imputation of genotypes called by GATK was higher than that by GATK and lower than that of the other methods. Interestingly, the correct-call rate among called genotypes of the Beagle imputation of genotypes called by GATK was lower than that by GATK, indicating that genotype imputation does not necessarily improve the accuracy of genotype calls, at least when preexisting high-quality reference panels are not available. The observation here is consistent with a recent finding that population-genetic parameters estimated via genotype imputation from low-coverage sequencing data are biased (Fu 2014).

**Table 3:** Comparison of the performance of the genotype-calling methods with human data

Method	Correct-Call Rate among Individuals (mean ± 2 SEM)	Correct-Call Rate among Called Genotypes (mean ± 2 SEM)
BGC	0.954 ± 0.0004	0.969 ± 0.0004
GATK	0.923 ± 0.0004	0.943 ± 0.0004
Samtools	0.966 ± 0.0004	0.966 ± 0.0004
ANGSD	0.967 ± 0.0004	0.967 ± 0.0004
GATK + Beagle	0.941 ± 0.0004	0.941 ± 0.0004

The correct-call rate among individuals is calculated among individuals with HapMap genotypes, where missing genotype calls are considered incorrect. On the other hand, the correct-call rate among called genotypes is calculated only among individuals with both HapMap genotypes and called genotypes, respectively.

**Figure 1:**
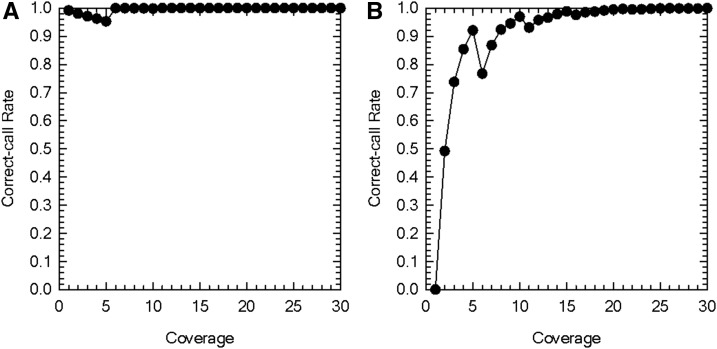
Correct-call rate of the high-coverage genotype caller as a function of the depth of coverage. (A) Correct-call rate when the true genotype of the individual is homozygous. (B) Correct-call rate when the true genotype of the individual is heterozygous. Error rate *ε* = 0.01. Results are based on a total of 10,000 simulation replications for each parameter set.

To examine when the depth of coverage is high enough to accurately call individual genotypes using the HGC, we examined the correct-call rate as a function of coverage when the true genotype is homozygous or heterozygous. Overall, the rate for correctly calling homozygotes is high (Figure 1A). It decreases with increased coverage when the coverage is five or less, and approaches one when the coverage is over 5. The overall rate for correctly calling heterozygotes increases with increased coverage, although it somewhat decreases when the coverage increases from five to six (Figure 1B). This decrease is consistent with the sudden increase in the rate for correctly calling homozygotes when the coverage increases from five to six. These patterns are observed because when two nucleotides have nonzero read counts, one of which has just one read, in the quartet, both of them are considered to be from an allele without error when the coverage is five or less, and the one with a single read count is considered to be due to a sequence error when the coverage is over five by HGC, as the likelihood that the single read is due to a sequence error becomes greater than that without error. In addition to the valley of the correct-call rate at coverage equal to six, there is another small valley at coverage equal to 11. This is because when two nucleotides have nonzero read counts, one of which has just two reads, in the quartet, both of them are considered to be from an allele without error when the coverage is <11, and the one with double reads is considered to be due to sequence errors when the coverage is ≥11 by HGC, as the likelihood that such reads are due to sequencing errors becomes greater than that without error.

To compare the performance of HGC in calling diploid genotypes at triallelic sites with that of other existing methods, we compared the correct-call rate of HGC to that of GATK and Samtools (Table 4). In addition, to examine the effect of incorrectly assuming biallelic polymorphisms on the accuracy of genotype calls at triallelic sites, we applied our BGC to the same data and examined its correct-call rate. The correct-call rate of HGC is higher than that by Samtools and slightly lower than that by GATK. Compared with genotype calls by Samtools, genotype calls by HGC and GATK quickly become more accurate with higher depths of coverage. The correct-call rate of BGC is the lowest. This is because BGC assumes at most two alleles, as many other methods including ANGSD do, and fails to correctly call genotypes containing the rarest allele. We confirmed that we correctly generated the simulated data by examining the realized parameter values in the population samples (Table S5).

**Table 4:** Performance of different genotype callers for calling diploid genotypes from nucleotide data at triallelic sites

Method	Mean Coverage	Correct-Call Rate (mean ± 2 SEM)	Correct-Call Rate among Called Genotypes (mean ± 2 SEM)
HGC	10	0.97 ± 0.00037	0.97 ± 0.00037
GATK	10	0.97 ± 0.00032	0.98 ± 0.00031
Samtools	10	0.96 ± 0.00040	0.96 ± 0.00040
BGC	10	0.78 ± 0.00099	0.79 ± 0.00099
HGC	15	0.99 ± 0.00022	0.99 ± 0.00022
GATK	15	1.00 ± 0.00013	1.00 ± 0.00013
Samtools	15	0.96 ± 0.00040	0.96 ± 0.00040
BGC	15	0.80 ± 0.00088	0.81 ± 0.00087
HGC	20	1.00 ± 0.00014	1.00 ± 0.00014
GATK	20	1.00 ± 0.00007	1.00 ± 0.00007
Samtools	20	0.97 ± 0.00031	0.97 ± 0.00031
BGC	20	0.80 ± 0.00084	0.81 ± 0.00083
HGC	30	1.00 ± 0.00005	1.00 ± 0.00005
GATK	30	1.00 ± 0.00002	1.00 ± 0.00002
Samtools	30	0.99 ± 0.00015	0.99 ± 0.00015
BGC	30	0.81 ± 0.00080	0.81 ± 0.00080

Allele frequencies *p*, *q*, and *r* are 0.7, 0.2, and 0.1, and the population is in Hardy–Weinberg equilibrium. Correct-call rate and that among called genotypes are calculated among *N* = 100 individuals and those with called genotypes, respectively. Error rate *ε* = 0.01. Results are based on a total of 10,000 simulation replications for each parameter set.

**Figure 2:**
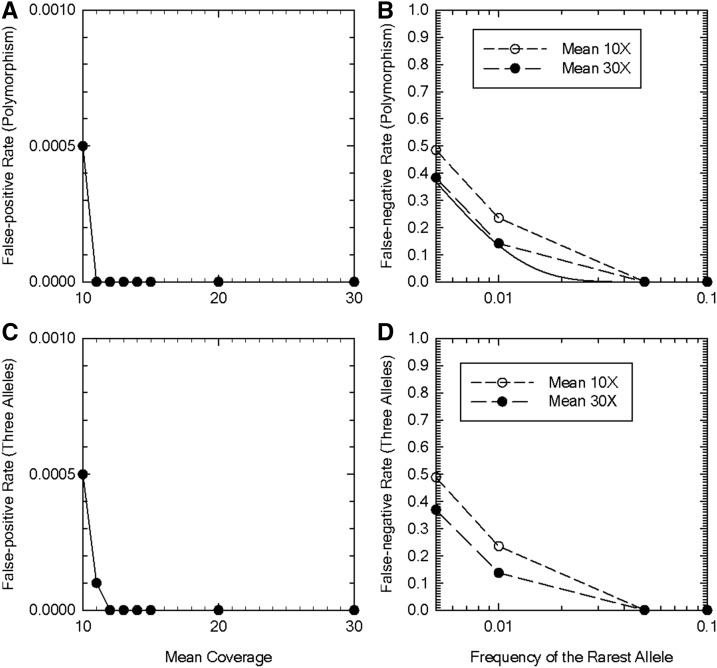
Power analysis of the high-coverage genotype caller. (A) False-positive rate of polymorphism detection as a function of the mean depth of coverage. (B) False-negative rate of detecting polymorphisms at biallelic sites as a function of the minor-allele frequency. The solid curve shows the theoretical minimum value as the probability that just one of the alleles is sampled in a finite sample of size *N* given by $q^{2N}+{\left(1-q\right)}^{2N},$ where *q* is the minor-allele frequency. (C) False-positive rate of detecting triallelic sites as a function of the mean depth of coverage with *q* = 0.1 (*i.e.*, the site is biallelic). (D) False-negative rate of inferring triallelic sites as a function of the frequency of the rarest allele. The frequency of the second most abundant allele is 0.2. The statistical significance of the likelihood-ratio tests is set at the 5% level in all panels. *N* = 100, error rate *ε* = 0.01, inbreeding coefficient *f* = 0 (Hardy–Weinberg equilibrium). Results are based on a total of 10,000 simulation replications for each parameter set.

To evaluate the power of HGC for detecting polymorphisms, we examined the false-positive and -negative rates as functions of the mean depth of coverage *μ*. The false-positive rate of detecting polymorphisms is low when *μ* is moderately high (10), and is essentially zero with μ>10 (Figure 2A). The false-negative rate of detecting polymorphisms is reasonably low when *μ* is moderately high (10), and approaches the theoretical minimum possible value, which is the probability that only one of the two alleles is sampled in a finite sample, when *μ* is 30 (Figure 2B). We note that the high false-negative rates with low minor-allele frequencies are mainly due to the finite sample size (100), which limits sampling rare alleles, and they remain high even with infinite coverage with any method. The power for detecting three alleles is essentially the same as that for detecting polymorphisms; the false-positive rate is low (Figure 2C) and false-negative rate is reasonably low (Figure 2D). These results indicate that HGC accurately describes polymorphisms with arbitrary numbers of alleles when the depth of coverage is high.

We applied the HGC to high-throughput sequencing data of 83 *D. pulex* clones from Kickapond to identify sites containing more than two alleles. We set the minimum coverage required to call a genotype of an individual at six so that the rate of falsely calling homozygotes as heterozygotes is low (Figure 1A). We identified a total of 4,403,303 significantly polymorphic sites at the 5% level from the sequence data filtered by multiple procedures to minimize analyzing misassembled regions and sites with mismapped reads. The vast majority (97.07%) of these were considered to contain two alleles. However, 127,167 (2.88%) and 2030 (0.05%) sites were considered to contain three and four alleles, respectively.

**Figure 3:**
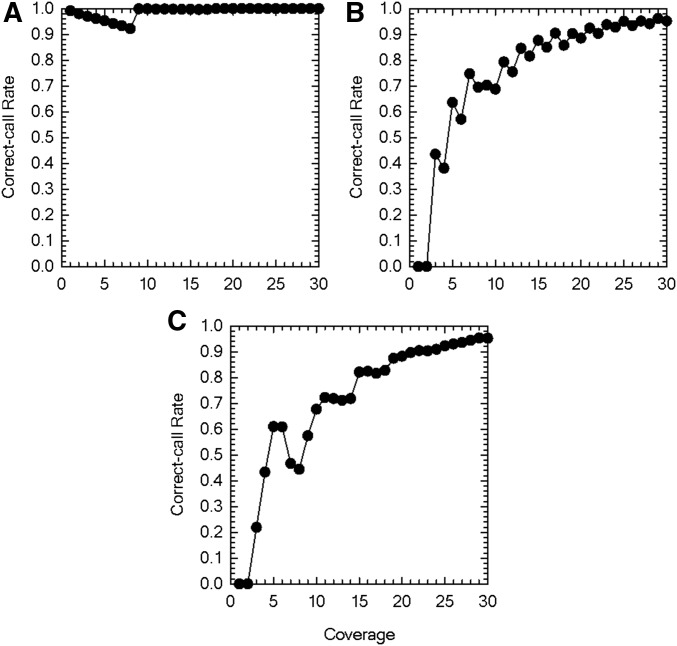
Correct-call rate of the triploid genotype caller as a function of the depth of coverage. (A) Correct-call rate when the true genotype of the individual is homozygous. (B) Correct-call rate when the true genotype of the individual is heterozygous containing two different nucleotides. (C) Correct-call rate when the true genotype of the individual is heterozygous containing three different nucleotides. Error rate *ε* = 0.01. Results are based on a total of 10,000 simulation replications for each parameter set.

The performance evaluation using computer simulations shows that calling accurate triploid genotypes requires much higher coverage than calling diploid genotypes (Figure 3). In particular, high coverage is needed to correctly call heterozygotes (Figure 3, B and C). This is because higher coverage is required to sequence all three chromosomes rather than just two chromosomes. Furthermore, it is difficult to distinguish the two alternative heterozygotes containing two alleles (*e.g.*, AAC *vs.* ACC) unless the coverage is very high. Overall, the correct-call rate increases with increased coverage for all three types of genotypes, although there are a few local exceptions. The valley of the rate for correctly calling homozygotes at coverage equal to eight (Figure 3A) is observed because when two nucleotides have nonzero read counts, one of which has just one read, in the quartet, both of them are considered to be from an allele without error when the coverage is eight or less, and the one with a single read is considered to be due to a sequence error when the coverage is over eight by the TRI, as the likelihood that the single read is due to a sequence error becomes greater than that without error. The sudden decrease in the rate for correctly calling heterozygotes containing three different nucleotides when coverage increases from six to seven (Figure 3C) is observed because when three nucleotides have nonzero read counts, one of which has just one read, in the quartet, all of them are considered to be from an allele without error when the coverage is six or less, and the one with a single read is considered to be due to a sequence error when the coverage is over six by the TRI, as the likelihood that the single read is due to a sequence error becomes greater than that without error. These observations highlight the inherent difficulty in calling polyploid genotypes not only for our method.

Comparison of the performance of our TRI to that of GATK shows that TRI yields more accurate called genotypes when the true genotype is a homozygote or heterozygote with two different nucleotides, whereas GATK yields more accurate called genotypes when the true genotype is a heterozygote with three different nucleotides (Table S6). Because the former two genotypes are generally far more abundant than the latter in natural populations, our method is likely to yield more accurate genotype calls than GATK when applied to real data. We also note that our method is more efficient than GATK in terms of both memory usage and computation time. TRI takes 2.749 sec whereas GATK takes 397.708 sec under the same computing environment to analyze simulated data with fixed coverage of 30 at 1,010,000 sites in an individual. This ∼145-fold difference in computation time becomes huge when analyzing organisms with a large genome size.

Because it takes additional time to prepare the input file of TRI from the BAM file, make a dictionary file of the reference, and add read groups to the BAM file to use GATK, we also compared the computation time between TRI and GATK taking these additional times into account. The additional times taken for the above analysis are 22.374 and 8.863 sec for TRI and GATK, respectively, making the total time 25.123 and 406.571 sec for TRI and GATK, respectively. Therefore, the total time taken for GATK is ∼16 times greater than that for TRI.

## Discussion

Given the rapid emergence of the field of population genomics, there is a need to systematically examine the performance of genotype callers. Recent studies (*e.g.*, Lynch 2009; Buerkle and Gompert 2013; Maruki and Lynch 2014) found that sequencing as many individuals as possible with low depths of coverage is the optimal strategy for estimating population-level parameters without calling genotypes with limited budgets. However, when depths of coverage are low, statistical uncertainty of individual sequence data are high and calling genotypes is difficult. Therefore, when inferring accurate genotypes is critical for a study, it becomes necessary to sequence a more limited number of individuals with higher depths of coverage.

In this study, we investigated how estimating genotype frequencies and error rates from a population sample and incorporating the population-level information into genotype-calling processes improves the performance of genotype calling, especially when the population deviates from HWE. We also examined the situation when individual coverage is high enough to accurately call genotypes without prior population-level estimates. To promote the use of our methods, we freely provide C++ programs implementing our genotype callers, along with their documentation (File S3, File S4, File S5, File S6, File S7, File S8, File S9, and File S10).

Consistent with previous results (Kim *et al.* 2011; Han *et al.* 2014), we find that allele-frequency estimates via genotype calling are biased, whereas those directly estimated from sequence read data are unbiased when depths of coverage are low. Given the importance of unbiased estimates of allele frequencies for subsequent population-genetic analyses, this reinforces the importance of estimating allele frequencies directly from sequence reads with low depths of coverage, as in Maruki and Lynch (2015). Our BGC takes advantage of prior population-level estimates of genotype frequencies and error rates to improve the accuracy of genotype calls in a population with an arbitrary mating system and internal population structure. The higher accuracy of called genotypes by BGC than obtained with currently widely used genotype callers in the majority of examined cases shows that our approach improves the ability to call genotypes while also providing unbiased estimates of allele frequencies.

Our performance evaluation of the HGC revealed that the rate of correctly calling homozygotes can be high if the minimum coverage required for calling genotypes is set at six or greater. By raising the minimum coverage cutoff to eight or greater, the rate for correctly calling heterozygotes can also be reasonably high. The power analyses of HGC for detecting polymorphisms and triallelic sites also indicate that the method is conservative and reasonably sensitive. The comparison of the performance of HGC to that of other existing methods showed that our method yields more accurate or as accurate diploid genotype calls at sites with more than two alleles.

The application of HGC to high-throughput sequencing data of 83 *Daphnia* clones from a population indicates that a non-negligible fraction of polymorphisms is triallelic and even tetra-allelic in *D. pulex*. There is growing evidence that triallelic polymorphisms exist in the human genome (*e.g.*, Hodgkinson and Eyre-Walker 2010; Nelson *et al.* 2012; Cao *et al.* 2015). In particular, a recent study sequencing many samples with high depths of coverage by Nelson *et al.* (2012) found that the fraction of triallelic sites is much higher than that previously expected. Furthermore, a recent study (Jenkins *et al.* 2014) found triallelic polymorphisms may be more informative for demographic inferences than biallelic polymorphisms. Therefore, it is important to relax the assumption of biallelic polymorphisms. Our method provides an efficient, flexible, and statistically rigorous framework for identifying polymorphic sites containing arbitrary numbers of alleles.

Because of its efficiency and flexibility, our method can be extended to analyses of population-genomic data from polyploid organisms. Our performance evaluation of the TRI revealed that much higher coverage is needed for correctly calling triploid genotypes than in the case of diploids. This conclusion is extended to genotype calling of organisms with higher ploidy, as the difficulties we found become greater. These results will help researchers to design sequencing strategies for population-genomic analyses of polyploid organisms.

As guidance on proper usage of the proposed four different genotype-calling methods, we provided a summary of the proposed methods (Table 5). BGC and HGC are both intended for calling diploid genotypes. Users should first apply HGC to population-genomic data of diploid organisms to identify sites with more than two alleles. Then, users should apply BGC to the data at biallelic sites, as BGC yields more accurate genotype calls than HGC at biallelic sites, unless the depth of coverage is extremely high. To facilitate this procedure, we provide a C++ program and documentation for setting the coverage of all individuals at zero at sites with more than two alleles as identified by HGC (File S11 and File S12). TRI and TET are extensions of HGC to triploid and tetraploid data, respectively. They enable highly efficient genotype calling at sites with arbitrary numbers of alleles. If future studies enable the population-level estimation of the genotype frequencies and error rates in diploid data at sites with arbitrary numbers of alleles and triploid and tetraploid data, we can, in principle, improve the accuracy of genotype calls from these data, although it will be very computationally expensive to estimate frequencies of many genotypes in these cases.

**Table 5:** Summary of the proposed methods

Method	Ploidy	Advantage	Disadvantage
BGC	Two	More accurate for biallelic SNPs	Assumes at most two alleles
HGC	Two	Highly efficient	Genotype-frequency information not used
		Arbitrary numbers of alleles	Less accurate for biallelic SNPs
TRI	Three	Highly efficient	Genotype-frequency information not used
		Arbitrary numbers of alleles	
TET	Four	Highly efficient	Genotype-frequency information not used
		Arbitrary numbers of alleles	

## Supplementary Material

Supplemental material is available online at www.g3journal.org/lookup/suppl/doi:10.1534/g3.117.039008/-/DC1.
