# Supplementary material for: Genotype Calling from Population-Genomic Sequencing Data
Source: G3 (Bethesda). 2017 Jan 19;7(5):1393–404. doi: 10.1534/g3.117.039008 (PMC5427492; doi:10.1534/g3.117.039008)
Supplement: Supplementary file 2 [file 1393FileS1.docx]

File S1. C++ program of the updated genotype-frequency estimator (GFE_v2.0.cpp). (.zip, 6 KB)

<http://www.g3journal.org/lookup/suppl/doi:10.1534/g3.117.039008/-/DC1/FileS1.zip>
