# Supplementary material for: Genotype Calling from Population-Genomic Sequencing Data
Source: G3 (Bethesda). 2017 Jan 19;7(5):1393–404. doi: 10.1534/g3.117.039008 (PMC5427492; doi:10.1534/g3.117.039008)
Supplement: Supplementary file 3 [file 1393FileS2.pdf]

GFE_v2.0.cpp

June 11, 2016

Author: Takahiro Maruki

C++ program of the genotype-frequency estimator (GFE)

This C++ program is for estimating allele and genotype frequencies from nucleotide read quartets (read counts of A, C, G, and T) derived from individual high-throughput sequencing data for multiple diploid individuals from a population by a maximum-likelihood (ML) method. For each site, ML estimates are obtained for the allele frequencies (under the assumption of no more than two alleles per site, these are by definition the two most abundant nucleotides in the population sample), error rate (due to all sources of error, not simply base quality), and disequilibrium/inbreeding coefficient. From the allele frequency and disequilibrium/inbreeding coefficient estimates, the program also estimates genotype frequencies. The statistical significance of the polymorphisms and their genotypic deviations from Hardy-Weinberg equilibrium (HWE) can also be tested, using the likelihood-ratio test statistics, with this software.

Input file. The input file is a tab-delimited text file, consisting of the reference nucleotide and individual nucleotide read quartets at every position. The first and second columns are the scaffold and position identifiers. The third column denotes the nucleotide of the reference sequence. Thereafter, the nucleotide read quartet is presented in each column per individual. Individual pro files in the 6-column format, which show the nucleotide quartet at each site, and the FASTA file (http://blast.ncbi.nlm.nih.gov/blastcgihelp.shtml) of the reference sequence, used for mapping sequence reads, are needed to make the input file. The individual pro files can be made from individual mpileup files (http://www.htslib.org/doc/samtools.html) using sam2pro written by Bernhard Haubold (http://guanine.evolbio.mpg.de/mlRho/sam2pro_0.8.tgz).

Output file. The output file is also a tab-delimited file, in this case consisting of 22 columns. Column: 1) scaffold (chromosome) identifier; 2) site identifier (coordinate); 3) nucleotide of the reference sequence; 4, 5) nucleotides of major and minor alleles; 6) depth of coverage in the population sample (sum of the coverage over the individuals); 7) effective number of sampled chromosomes; 8) number of individuals with at least one read; 9, 10) ML estimates of the major and minor allele counts; 11, 12) ML estimates of the major and minor allele frequencies (p and q); 13, 14) ML estimates of the error rate under the full model and null model assuming monomorphism; 15) ML estimate of the disequilibrium coefficient; 16) ML estimate of the inbreeding coefficient; 17, 18, 19) ML estimates of the frequencies of major homozygotes, heterozygotes, and minor homozygotes; 20) ML estimate of the per-site heterozygosity ($2pq$); 21) likelihood-ratio test statistic for polymorphism; 22) likelihood-ratio test statistic for HWE deviation. The likelihood-ratio test statistics for polymorphism and HWE-deviation are expected

to asymptotically correspond to chi-squared distributions with two and one degrees of freedom, respectively.

Reference

If you use this program, please cite the following paper:

Maruki, T., and Lynch, M., (2015) Genotype-frequency estimation from high-throughput sequencing data. *Genetics* **201**:473-486.

Instructions

Below are specific procedures for using the program:

1. Make the input file using Package-GFE (https://github.com/Takahiro-Maruki/Package-GFE).

2. Compile the program using the following command:

g++ -o GFE_v2.0 GFE_v2.0.cpp -lm

3. Run the program by typing the following command:

./GFE_v2.0 -in In_GFE.txt -out Out_GFE.txt

- In_GFE.txt and Out_GFE.txt are default names of the input and output files, respectively. The input and output file names can be specified by adding the `-in' and `-out' options, respectively.

- The minimum required coverage and maximum allowed coverage for each individual can be specified by adding the `-min_cov' and `-max_cov' options, respectively. Their default values are 1 and 2,000,000,000, respectively.

- The input file of the LD estimator (Maruki and Lynch 2014) can be prepared by specifying the `l' mode. The output in this mode is conditioned on significant polymorphisms.

- The input file of the Bayesian genotype caller (BGC) can be prepared by specifying the mode as `c'. The output in this mode is also conditioned on significant polymorphisms.

- The chi-square critical value for the polymorphism test can be specified by adding the `-cv' option. The default critical value is 5.991 (at the 5% level).

- A usage help message explaining these options can be shown by typing the following command:

./GFE_v2.0 –h

Updates from the previous version (GFE)

• Options for specifying the minimum and maximum coverage for each individual added.

• A warning message printed out on the screen when the identity of the minor allele is ambiguous.

• `c’ mode added to prepare the input file of the Bayesian genotype caller (BGC).

Copyright notice

This program is freely available; and can be redistributed and/or modified under the terms of the GNU General Public License as published by the Free Software Foundation; either version 2 of the License, or (at your option) any later version.

This program is distributed in the hope that it will be useful, but WITHOUT ANY WARRANTY; without even the implied warranty of MERCHANTABILITY or FITNESS FOR A PARTICULAR PURPOSE. See the GNU General Public License for more details.

For a copy of the GNU General Public License write to the Free Software Foundation, Inc., 59 Temple Place, Suite 330, Boston, MA 02111-1307 USA

Contact

If you have difficulty using this software, please send the following information to Takahiro Maruki (tmaruki@indiana.edu):

1. Brief explanation of the problem.

2. Command entered.

3. Part of the input file.

4. Part of the output file.
