# Supplementary material for: Genotype Calling from Population-Genomic Sequencing Data
Source: G3 (Bethesda). 2017 Jan 19;7(5):1393–404. doi: 10.1534/g3.117.039008 (PMC5427492; doi:10.1534/g3.117.039008)
Supplement: Supplementary file 6 [file 1393FileS4.pdf]

BGC.cpp

January 18, 2017

Author: Takahiro Maruki

C++ program of a Bayesian genotype caller for diploid organisms useful for calling genotypes from low-coverage sequencing data

This C++ program is for calling genotypes from nucleotide-read quartets (read counts of A, C, G, and T) derived from individual high-throughput sequencing data for multiple diploid individuals from a population by a maximum-likelihood (ML) method. This genotype caller incorporates the genotype-frequency and sequencing-error rate estimates predetermined by an ML genotype-frequency estimator (GFE) (Maruki and Lynch 2015) to improve the accuracy of genotypes called from low-coverage sequencing data. At each of the significantly polymorphic sites pre-identified by GFE, the genotype for each individual is estimated by maximizing the likelihood of the observed data.

Input file. The input file is a tab-delimited text file, and can be prepared using GFE_v2.0.cpp, specifying the mode as 'c`. The meanings of the first twelve columns are: 1) scaffold (chromosome) identifier; 2) site identifier (coordinate); 3) nucleotide of the reference sequence; 4, 5) nucleotides of the major and minor alleles, respectively (1: A, 2: C, 3: G, 4: T); 6) depth of coverage in the population sample (sum of the coverage over the individuals; 7) sequencing-error rate estimate; 8, 9, 10) ML estimates of the frequencies of major homozygotes, heterozygotes, and minor homozygotes, respectively; 11) likelihood-ratio test statistic for polymorphism; 12) likelihood-ratio test statistic for deviation from Hardy-Weinberg equilibrium. Thereafter, nucleotide-read quartets are shown for each individual in each of the columns.

Output file. The output file is also a tab-delimited file. The meanings of the first five columns are: 1) scaffold (chromosome) identifier; 2) site identifier (coordinate); 3) major-allele frequency; 4) sequencing-error rate estimate; 5) depth of coverage in the population sample (sum of the coverage over the individuals. Thereafter, the called genotype is shown for each individual in each of the columns.

Reference

If you use this program, please cite the following paper:

Maruki, T., and Lynch, M., (in press) Genotype calling from population-genomic sequencing data. *G3: Genes | Genomes | Genetics*.

Instructions

Below are specific procedures for using the program:

1. Make the input file, using GFE_v2.0 in the `c' mode.

2. Compile the program.by typing the following command:

g++ -o BGC BGC.cpp -lm

3. Run the program by typing the following command:

./BGC -in In_BGC.txt -out Out_BGC.txt

- In_BGC.txt and Out_BGC.txt are default names of the input and output files, respectively. The input and output file names can be specified by adding the `-in' and `-out' options, respectively.

- The minimum required coverage and maximum allowed coverage to call a genotype of an individual can be specified by adding the `-min_cov' and `-max_cov' options, respectively. Their default values are 1 and 2,000,000,000, respectively.

- The posterior probabilities of the genotypes for each individual can be shown in the output by adding the `-gp' option. When this value is set at one, the posterior probabilities of the major and minor homozygotes separated by a slash are shown for each individual.

- A usage help message explaining these options can be shown by typing the following command:

./BGC –h

Copyright notice

This program is freely available; and can be redistributed and/or modified under the terms of the GNU General Public License as published by the Free Software Foundation; either version 2 of the License, or (at your option) any later version.

This program is distributed in the hope that it will be useful, but WITHOUT ANY WARRANTY; without even the implied warranty of MERCHANTABILITY or FITNESS FOR A PARTICULAR PURPOSE. See the GNU General Public License for more details.

For a copy of the GNU General Public License write to the Free Software Foundation, Inc., 59 Temple Place, Suite 330, Boston, MA 02111-1307 USA

Contact

If you have difficulty using this software, please send the following information to Takahiro Maruki (tmaruki@indiana.edu):

1. Brief explanation of the problem.

2. Command entered.

3. Part of the input file.

4. Part of the output file.
