# Supplementary material for: Genotype Calling from Population-Genomic Sequencing Data
Source: G3 (Bethesda). 2017 Jan 19;7(5):1393–404. doi: 10.1534/g3.117.039008 (PMC5427492; doi:10.1534/g3.117.039008)
Supplement: Supplementary file 9 [file 1393FileS6.pdf]

HGC.cpp

January 18, 2017

Author: Takahiro Maruki

C++ program for calling genotypes from high-coverage high-throughput sequencing data of diploid organisms

This C++ program is for calling genotypes from nucleotide read quartets (read counts of A, C, G, and T) derived from individual high-throughput sequencing data for multiple diploid individuals from a population by a maximum-likelihood (ML) method. Although this program can be used to call genotypes from low-coverage sequencing data, moderately high coverage is needed to call accurate genotypes with this program. At each site, the genotype and sequence-error rate for each individual are estimated by maximizing the likelihood of the observed data. To avoid analyzing false polymorphisms, number of alleles at the site is estimated based on significant genotypes with respect to the genotype homozygous for the most abundant nucleotide in the population sample. We use a likelihood-ratio test, which is expected to be asymptotically chi-square distributed with one degree of freedom, to examine the statistical significance.

Input file. The input file is a tab-delimited text file, consisting of the reference nucleotide and individual nucleotide read quartets at each position. The format is the same as that for our genotype-frequency estimator (Maruki and Lynch 2015), and can be made using our software package GFE (https://github.com/Takahiro-Maruki/Package-GFE). The first and second columns are the scaffold and position identifiers. The third column denotes the nucleotide of the reference sequence. Thereafter, the nucleotide read quartet for each individual is presented in each of the columns.

Output file. The output file is also a tab-delimited file. The meanings of the first eleven columns are: 1) scaffold (chromosome) identifier; 2) site identifier (coordinate); 3) nucleotide of the reference sequence; 4) number of alleles; 5) identity of the major allele; 6) identity of the minor allele; 7) depth of coverage in the population sample (sum of the coverage over the individuals); 8) number of called genotypes; 9) count of the major allele; 10) count of the minor allele; 11) mean error-rate estimate among called genotypes. Next, the called genotypes are shown for each individual in each of the columns. The last four columns show the maximum values of the likelihood-ratio test statistics of called genotypes containing A, C, G, and T, respectively. These are expected to be asymptotically chi-square distributed with one degree of freedom.

Reference

If you use this program, please cite the following paper:

Maruki, T., and Lynch, M., (in press) Genotype calling from population-genomic sequencing data. *G3: Genes | Genomes | Genetics*.

Instructions

Below are specific procedures for using the program:

1. Make the input file using GFE (https://github.com/Takahiro-Maruki/Package-GFE).

2. Compile the program.by typing the following command:

g++ -o HGC HGC.cpp -lm

3. Run the program using the following command:

./HGC -in In_HGC.txt -out Out_HGC.txt

- In_HGC.txt and Out_HGC.txt are default names of the input and output files, respectively. The input and output file names can be specified by adding the `-in' and `-out' options, respectively.

- The chi-square critical value for the polymorphism test can be specified by adding the `-cv' option

- The minimum required coverage and maximum allowed coverage to call a genotype of an individual can be specified by adding the `-min_cov' and `-max_cov' options, respectively. Their default values are 1 and 2,000,000,000, respectively.

- The maximum allowed error rate per read per site can be specified by adding the `-max_e' option.

- A usage help message explaining these options can be shown by typing the following command:

./HGC –h

Copyright notice

This program is freely available; and can be redistributed and/or modified under the terms of the GNU General Public License as published by the Free Software Foundation; either version 2 of the License, or (at your option) any later version.

This program is distributed in the hope that it will be useful, but WITHOUT ANY WARRANTY; without even the implied warranty of MERCHANTABILITY or FITNESS FOR A PARTICULAR PURPOSE. See the GNU General Public License for more details.

For a copy of the GNU General Public License write to the Free Software Foundation, Inc., 59 Temple Place, Suite 330, Boston, MA 02111-1307 USA

Contact

If you have difficulty using this software, please send the following information to Takahiro Maruki (tmaruki@indiana.edu):

1. Brief explanation of the problem.
2. Command entered.
3. Part of the input file.
4. Part of the output file.
