# Supplementary material for: Genotype Calling from Population-Genomic Sequencing Data
Source: G3 (Bethesda). 2017 Jan 19;7(5):1393–404. doi: 10.1534/g3.117.039008 (PMC5427492; doi:10.1534/g3.117.039008)
Supplement: Supplementary file 18 [file 1393FileS12.pdf]

Rem_Multi_Allelic.cpp

January 18, 2017

Author: Takahiro Maruki

C++ program for removing data at sites with more than two alleles

This C++ program is for setting the depth of coverage of all individuals at zero at sites with more than two alleles identified by the high-coverage genotype caller (HGC) in the file of nucleotide-read quartets (counts of A, C, G, and T) of individuals in the population. The output file can be used as the input file of the genotype-frequency estimator (GFE_v2.0), which can prepare the input file of the Bayesian genotype caller (BGC).

Input files. This program requires two input files. One is the tab-delimited text file of nucleotide-read quartets of individuals in the population. Its format is the same as that for our genotype-frequency estimator (Maruki and Lynch 2015), and can be made using our software package GFE (https://github.com/Takahiro-Maruki/Package-GFE). The first and second columns are the scaffold and position identifiers. The third column denotes the nucleotide of the reference sequence. Thereafter, the nucleotide read quartet for each individual is presented in each of the columns. The other is the tab-delimited text file of the list of sites with more than two alleles identified by HGC, which consists of two columns. Column: 1) scaffold (chromosome) identifier; 2) site identifier (coordinate). This file can be easily prepared from the output of HGC, for example, by typing the following command:

```
awk -v OFS='\t' '{if ($1 == "scaffold" || $4 != "NA" && $4 > 2) print $1, $2}' Out_HGC.txt >
List_MA.txt
```

Output file. The format of the output file is identical to that of the input file. Here, the coverage of all individuals is set at zero at sites with more than two alleles identified by HGC.

Reference

If you use this program, please cite the following paper:

Maruki, T., and Lynch, M., (in press) Genotype calling from population-genomic sequencing data. *G3: Genes | Genomes | Genetics*.

Instructions

Below are specific procedures for using the program:

1. Make the input file using Package-GFE (https://github.com/Takahiro-Maruki/Package-GFE).

2. Compile the program using the following command:

g++ -o Rem_Multi_Allelic Rem_Multi_Allelic.cpp -lm

3. Run the program by typing the following command:

./ Rem_Multi_Allelic -pf pro.txt -mf List_MA.txt -out Out_RMA.txt

- pro.txt, List_MA.txt, and Out_RMA.txt are default names of the input pro file, input file of the list of sites with more than two alleles, and output file, respectively. Their file names can be specified by adding the `-pf' `-mf', and `-out' options, respectively.

- A usage help message explaining these options can be shown by typing the following command:

./ Rem_Multi_Allelic -h

Copyright notice

This program is freely available; and can be redistributed and/or modified under the terms of the GNU General Public License as published by the Free Software Foundation; either version 2 of the License, or (at your option) any later version.

This program is distributed in the hope that it will be useful, but WITHOUT ANY WARRANTY; without even the implied warranty of MERCHANTABILITY or FITNESS FOR A PARTICULAR PURPOSE. See the GNU General Public License for more details.

For a copy of the GNU General Public License write to the Free Software Foundation, Inc., 59 Temple Place, Suite 330, Boston, MA 02111-1307 USA

Contact

If you have difficulty using this software, please send the following information to Takahiro Maruki (tmaruki@indiana.edu):

1. Brief explanation of the problem.

2. Command entered.

3. Part of the input file.

4. Part of the output file.
