# Supplementary material for: Genotype Calling from Population-Genomic Sequencing Data
Source: G3 (Bethesda). 2017 Jan 19;7(5):1393–404. doi: 10.1534/g3.117.039008 (PMC5427492; doi:10.1534/g3.117.039008)
Supplement: Supplementary file 19 [file 1393TableS1.docx]

**TABLE S1** **Probability of an observed nucleotide read as a function of the individual genotype *g* and error rate *ε* in the triploid sequence data**.

| Genotype | Nucleotide read | | | | |  |
| --- | --- | --- | --- | --- | --- | --- |
|  | A | C | | G | | T |
| AAA | 1 - *ϵ* | | ϵ/3 | | ϵ/3 | ϵ/3 |

CCC ϵ/3 1 - ϵ ϵ/3 ϵ/3

GGG ϵ/3 ϵ/3 1 - ϵ ϵ/3

TTT ϵ/3 ϵ/3 ϵ/3 1 - ϵ

ACC (1/3) – (ϵ/9) (2/3) - (5ϵ/9) ϵ/3 ϵ/3

AGG (1/3) – (ϵ/9) ϵ/3 (2/3) - (5ϵ/9) ϵ/3

ATT (1/3) – (ϵ/9) ϵ/3 ϵ/3 (2/3) - (5ϵ/9)

CGG ϵ/3 (1/3) - (ϵ/9) (2/3) - (5ϵ/9) ϵ/3

CTT ϵ/3 (1/3) - (ϵ/9) ϵ/3 (2/3) - (5ϵ/9)

GTT ϵ/3 ϵ/3 (1/3) - (ϵ/9) (2/3) - (5ϵ/9)

AAC (2/3) - (5ϵ/9) (1/3) – (ϵ/9) ϵ/3 ϵ/3

AAG (2/3) - (5ϵ/9) ϵ/3 (1/3) - (ϵ/9) ϵ/3

AAT (2/3) - (5ϵ/9) ϵ/3 ϵ/3 (1/3) - (ϵ/9)

CCG ϵ/3 (2/3) - (5ϵ/9) (1/3) – (ϵ/9) ϵ/3

CCT ϵ/3 (2/3) - (5ϵ/9) ϵ/3 (1/3) – (ϵ/9)

GGT ϵ/3 ϵ/3 (2/3) - (5ϵ/9) (1/3) – (ϵ/9)

ACG (1/3) - (ϵ/9) (1/3) - (ϵ/9) (1/3) - (ϵ/9) ϵ/3

ACT (1/3) - (ϵ/9) (1/3) - (ϵ/9) ϵ/3 (1/3) - (ϵ/9)

AGT (1/3) – (ϵ/9) ϵ/3 (1/3) - (ϵ/9) (1/3) - (ϵ/9)

CGT ϵ/3 (1/3) - (ϵ/9) (1/3) - (ϵ/9) (1/3) - (ϵ/9)
