# Supplementary material for: Genotype Calling from Population-Genomic Sequencing Data
Source: G3 (Bethesda). 2017 Jan 19;7(5):1393–404. doi: 10.1534/g3.117.039008 (PMC5427492; doi:10.1534/g3.117.039008)
Supplement: Supplementary file 20 [file 1393TableS2.docx]

**TABLE S2** **Probability of an observed nucleotide read as a function of the individual genotype *g* and error rate *ε* in the tetraploid sequence data**.

| Genotype | Nucleotide read | | | | |  |
| --- | --- | --- | --- | --- | --- | --- |
|  | A | C | | G | | T |
| AAAA | 1 - *ϵ* | | ϵ/3 | | ϵ/3 | ϵ/3 |

CCCC ϵ/3 1 - ϵ ϵ/3 ϵ/3

GGGG ϵ/3 ϵ/3 1 - ϵ ϵ/3

TTTT ϵ/3 ϵ/3 ϵ/3 1 - ϵ

ACCC 1/4 (3/4) - (2ϵ/3) ϵ/3 ϵ/3

AGGG 1/4 ϵ/3 (3/4) - (2ϵ/3) ϵ/3

ATTT 1/4 ϵ/3 ϵ/3 (3/4) - (2ϵ/3)

CGGG ϵ/3 1/4 (3/4) - (2ϵ/3) ϵ/3

CTTT ϵ/3 1/4 ϵ/3 (3/4) - (2ϵ/3)

GTTT ϵ/3 ϵ/3 1/4 (3/4) - (2ϵ/3)

AACC (1/2) - (ϵ/3) (1/2) - (ϵ/3) ϵ/3 ϵ/3

AAGG (1/2) - (ϵ/3) ϵ/3 (1/2) - (ϵ/3) ϵ/3

AATT (1/2) - (ϵ/3) ϵ/3 ϵ/3 (1/2) - (ϵ/3)

CCGG ϵ/3 (1/2) - (ϵ/3) (1/2) - (ϵ/3) ϵ/3

CCTT ϵ/3 (1/2) - (ϵ/3) ϵ/3 (1/2) - (ϵ/3)

GGTT ϵ/3 ϵ/3 (1/2) - (ϵ/3) (1/2) - (ϵ/3)

AAAC (3/4) - (2ϵ/3) 1/4 ϵ/3 ϵ/3

AAAG (3/4) - (2ϵ/3) ϵ/3 1/4 ϵ/3

AAAT (3/4) - (2ϵ/3) ϵ/3 ϵ/3 1/4

CCCG ϵ/3 (3/4) - (2ϵ/3) 1/4 ϵ/3

CCCT ϵ/3 (3/4) - (2ϵ/3) ϵ/3 1/4

GGGT ϵ/3 ϵ/3 (3/4) - (2ϵ/3) 1/4

AACG (1/2) - (ϵ/3) 1/4 1/4 ϵ/3

AACT (1/2) - (ϵ/3) 1/4 ϵ/3 1/4

AAGT (1/2) - (ϵ/3) ϵ/3 1/4 1/4

ACCG 1/4 (1/2) - (ϵ/3) 1/4 ϵ/3

ACCT 1/4 (1/2) - (ϵ/3) ϵ/3 1/4

CCGT ϵ/3 (1/2) - (ϵ/3) 1/4 1/4

ACGG 1/4 1/4 (1/2) - (ϵ/3) ϵ/3

AGGT 1/4 ϵ/3 (1/2) - (ϵ/3) 1/4

CGGT ϵ/3 1/4 (1/2) - (ϵ/3) 1/4

ACTT 1/4 1/4 ϵ/3 (1/2) - (ϵ/3)

AGTT 1/4 ϵ/3 1/4 (1/2) - (ϵ/3)

CGTT ϵ/3 1/4 1/4 (1/2) - (ϵ/3)

ACGT 1/4 1/4 1/4 1/4
