# Supplementary material for: Genotype Calling from Population-Genomic Sequencing Data
Source: G3 (Bethesda). 2017 Jan 19;7(5):1393–404. doi: 10.1534/g3.117.039008 (PMC5427492; doi:10.1534/g3.117.039008)
Supplement: Supplementary file 21 [file 1393TableS3.docx]

**TABLE S3 Comparison of the allele-frequency estimates and called genotypes by different methods with moderately high depths of coverage.**

Correct-call Rate Correct-call Rate

among Individuals among Called Genotypes

Method  *q* *f* $\hat{q}$ (Mean ± 2SE) (Mean ± 2SE) (Mean ± 2SE)

Proposed 0.1 0 (HWE) 0.10 ± 0.00044 0.99 ± 0.00018 0.99 ± 0.00017

GATK 0.1 0 (HWE) 0.10 ± 0.00042 0.99 ± 0.00019 0.99 ± 0.00019

Samtools 0.1 0 (HWE) 0.11 ± 0.00045 0.97 ± 0.00033 0.97 ± 0.00033

ANGSD 0.1 0 (HWE) 0.10 ± 0.00044 0.99 ± 0.00019 0.99 ± 0.00019

Proposed 0.1 minimized 0.10 ± 0.00041 0.99 ± 0.00018 0.99 ± 0.00017

GATK 0.1 minimized 0.10 ± 0.00041 0.99 ± 0.00020 0.99 ± 0.00020

Samtools 0.1 minimized 0.11 ± 0.00043 0.97 ± 0.00033 0.97 ± 0.00033

ANGSD 0.1 minimized 0.10 ± 0.00041 0.99 ± 0.00019 0.99 ± 0.00019

Proposed 0.1 maximized 0.10 ± 0.00059 1.00 ± 0.00005 1.00 ± 0.00005

GATK 0.1 maximized 0.10 ± 0.00059 1.00 ± 0.00010 1.00 ± 0.00010

Samtools 0.1 maximized 0.11 ± 0.00060 0.97 ± 0.00036 0.97 ± 0.00036

ANGSD 0.1 maximized 0.10 ± 0.00060 0.99 ± 0.00020 0.99 ± 0.00020

Proposed 0.3 0 (HWE) 0.30 ± 0.00066 0.99 ± 0.00024 0.99 ± 0.00023

GATK 0.3 0 (HWE) 0.29 ± 0.00066 0.98 ± 0.00030 0.98 ± 0.00030

Samtools 0.3 0 (HWE) 0.31 ± 0.00065 0.98 ± 0.00031 0.98 ± 0.00031

ANGSD 0.3 0 (HWE) 0.30 ± 0.00066 0.99 ± 0.00024 0.99 ± 0.00024

Proposed 0.3 minimized 0.30 ± 0.00050 0.99 ± 0.00024 0.99 ± 0.00021

GATK 0.3 minimized 0.29 ± 0.00050 0.97 ± 0.00034 0.97 ± 0.00034

Samtools 0.3 minimized 0.31 ± 0.00049 0.98 ± 0.00029 0.98 ± 0.00029

ANGSD 0.3 minimized 0.30 ± 0.00050 0.99 ± 0.00024 0.99 ± 0.00024

Proposed 0.3 maximized 0.30 ± 0.00091 1.00 ± 0.00004 1.00 ± 0.00004

GATK 0.3 maximized 0.30 ± 0.00091 0.99 ± 0.00016 0.99 ± 0.00016

Samtools 0.3 maximized 0.31 ± 0.00090 0.97 ± 0.00036 0.97 ± 0.00036

ANGSD 0.3 maximized 0.30 ± 0.00092 0.98 ± 0.00025 0.98 ± 0.00025

*q,* $\hat{q}$, and *f* are the minor-allele frequency, its estimate, and inbreeding coefficient, respectively. $\hat{q}$ by the proposed method and ANGSD are directly estimated from sequence-read data by the genotype-frequency estimator (Maruki and Lynch 2015) and Kim *et al.*’s method (2011), respectively. Called genotypes by the proposed method are by the Bayesian genotype caller. The correct-call rate is a fraction of individuals with correctly called genotypes among *N* = 100 individuals, where missing genotype calls are considered incorrect. On the other hand, the correct-call rate among called genotypes is calculated only among individuals with called genotypes. SE denotes standard error of the mean. Mean depth of coverage *µ* = 10, error rate *ε* = 0.01. Results are based on a total of 10,000 simulation replications for each parameter set.
