# Supplementary material for: Genotype Calling from Population-Genomic Sequencing Data
Source: G3 (Bethesda). 2017 Jan 19;7(5):1393–404. doi: 10.1534/g3.117.039008 (PMC5427492; doi:10.1534/g3.117.039008)
Supplement: Supplementary file 22 [file 1393TableS4.docx]

**TABLE S4** Summary of the realized parameter values in population samples in simulated diploid data at biallelic sites

Mean Coverage $\gamma_{1}$ $\gamma_{3}$ Sample $\gamma_{1}$ (Mean ± SD) Sample $\gamma_{3}$ (Mean ± SD) Sample *ε* (Mean ± SD)

3 0.81 0.01 0.81 ± 0.039 0.01 ± 0.010 0.01 ± 0.006

3 0.8 0 0.80 ± 0.040 0.00 ± 0.000 0.01 ± 0.006

3 0.9 0.1 0.90 ± 0.030 0.10 ± 0.030 0.01 ± 0.006

3 0.49 0.09 0.49 ± 0.050 0.09 ± 0.029 0.01 ± 0.006

3 0.4 0 0.40 ± 0.049 0.00 ± 0.000 0.01 ± 0.006

3 0.7 0.3 0.70 ± 0.046 0.30 ± 0.046 0.01 ± 0.006

10 0.81 0.01 0.81 ± 0.040 0.01 ± 0.010 0.01 ± 0.003

10 0.8 0.0 0.80 ± 0.040 0.00 ± 0.000 0.01 ± 0.003

10 0.9 0.1 0.90 ± 0.030 0.10 ± 0.030 0.01 ± 0.003

10 0.49 0.09 0.49 ± 0.050 0.09 ± 0.030 0.01 ± 0.003

10 0.4 0 0.40 ± 0.049 0.00 ± 0.000 0.01 ± 0.003

10 0.7 0.3 0.70 ± 0.046 0.30 ± 0.046 0.01 ± 0.003

$\gamma_{1}$ and $\gamma_{3}$ are frequencies of major and minor homozygotes, respectively. SD denotes standard deviation of the mean. Sample size *N* = 100, error rate *ϵ* = 0.01. Results are based on a total of 10,000 simulation replications for each parameter set.
