# Supplementary material for: Genotype Calling from Population-Genomic Sequencing Data
Source: G3 (Bethesda). 2017 Jan 19;7(5):1393–404. doi: 10.1534/g3.117.039008 (PMC5427492; doi:10.1534/g3.117.039008)
Supplement: Supplementary file 23 [file 1393TableS5.docx]

**TABLE S5** Summary of the realized parameter values in population samples in simulated diploid data at triallelic sites

Mean Coverage Sample *p* (Mean ± SD) Sample *q* (Mean ± SD) Sample *r* (Mean ± SD) Sample *ϵ* (Mean ± SD)

10 0.70 ± 0.033 0.20 ± 0.029 0.10 ± 0.022 0.01 ± 0.003

15 0.70 ± 0.033 0.20 ± 0.028 0.10 ± 0.021 0.01 ± 0.003

20 0.70 ± 0.033 0.20 ± 0.028 0.10 ± 0.021 0.01 ± 0.002

30 0.70 ± 0.032 0.20 ± 0.028 0.10 ± 0.021 0.01 ± 0.002

*p*, *q*, and *r* are frequencies of the most abundant, second most abundant, and rarest alleles, respectively. SD denotes standard deviation of the mean. Sample size *N* = 100, error rate *ϵ* = 0.01. Results are based on a total of 10,000 simulation replications for each parameter set.
