# Supplementary material for: Genotype Calling from Population-Genomic Sequencing Data
Source: G3 (Bethesda). 2017 Jan 19;7(5):1393–404. doi: 10.1534/g3.117.039008 (PMC5427492; doi:10.1534/g3.117.039008)
Supplement: Supplementary file 24 [file 1393TableS6.docx]

**TABLE S6 Comparison of the performance of the triploid genotype caller to that of GATK**

Method Coverage Genotype Correct-call Rate Correct-call Rate

among Called Genotypes

TRI 10 Homozygote 0.999 0.999

GATK 10 Homozygote 0.963 0.963

TRI 10 Heterozygote with two different nucleotides 0.685 0.786

GATK 10 Heterozygote with two different nucleotides 0.770 0.770

TRI 10 Heterozygote with three different nucleotides 0.665 0.678

GATK 10 Heterozygote with three different nucleotides 0.765 0.765

TRI 20 Homozygote 1.000 1.000

GATK 20 Homozygote 0.997 0.997

TRI 20 Heterozygote with two different nucleotides 0.881 0.926

GATK 20 Heterozygote with two different nucleotides 0.904 0.904

TRI 20 Heterozygote with three different nucleotides 0.892 0.901

GATK 20 Heterozygote with three different nucleotides 0.977 0.977

TRI 30 Homozygote 1.000 1.000

GATK 30 Homozygote 0.996 0.996

TRI 30 Heterozygote with two different nucleotides 0.953 0.973

GATK 30 Heterozygote with two different nucleotides 0.958 0.958

TRI 30 Heterozygote with three different nucleotides 0.952 0.952

GATK 30 Heterozygote with three different nucleotides 0.998 0.998

Correct-call rate and that among called genotypes are calculated among 10,000 simulation replications and those with called genotypes, respectively. Error rate *ε* = 0.01.
